# Notoginsenoside‐R1 ameliorates palmitic acid‐induced insulin resistance and oxidative stress in HUVEC via Nrf2/ARE pathway

**DOI:** 10.1002/fsn3.3696

**Published:** 2023-10-18

**Authors:** Jingjing Wang, Xun He, Shiwen Lv

**Affiliations:** ^1^ Department of Pharmacy, Affiliated Jinhua Hospital Zhejiang University School of Medicine Jinhua China

**Keywords:** endothelium, insulin resistance, notoginsenoside‐R1, Nrf2, oxidative stress, *Panax notoginseng*

## Abstract

*Panax notoginseng*, a Chinese traditional food and herb medicine, possesses notable cardiovascular health‐promoting properties, with notoginsenoside (NG)‐R1 being a key active compound. Insulin resistance represents a global health concern associated with various metabolic disorders. This study investigated the effects of NG‐R1 on palmitic acid (PA)‐induced insulin resistance and oxidative stress in human umbilical vein endothelial cells (HUVECs). Our findings demonstrate that NG‐R1 significantly alleviated impaired glucose uptake, enhanced the phosphorylation of protein kinase B (PKB/Akt) at Ser473, and reduced the phosphorylation of insulin receptor substrate 1 (IRS‐1) at Ser307 in PA‐treated HUVECs. Furthermore, NG‐R1 treatment significantly lowered the levels of malondialdehyde (MDA) and 4‐hydroxynonenal (4‐HNE), while increasing the ratio of reduced glutathione (GSH) to oxidized glutathione (GSSG). Additionally, NG‐R1 activated the Nrf2/ARE signaling pathway, leading to a substantial increase in the expression of antioxidant enzymes. Notably, knockdown of Nrf2 attenuated the beneficial effects of NG‐R1 on PA‐induced insulin resistance and oxidative stress in HUVECs, suggesting that NG‐R1 exerts its effects through the Nrf2/ARE pathway. In summary, our study reveals that NG‐R1 ameliorated PA‐induced insulin resistance in HUVECs via Nrf2/ARE pathway, providing novel insights into its potential for alleviating metabolic disorders and cardiovascular disease.

## Graphical abstract

NG‐R1 ameliorated PA‐induced insulin resistance in HUVECs via Nrf2/ARE pathway, which provides novel insights into the alleviation of metabolic diseases and cardiovascular disease.

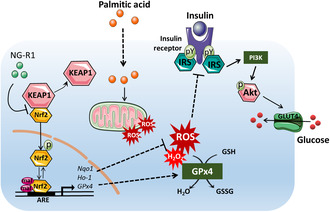

## 1 INTRODUCTION

**FIGURE 1:**
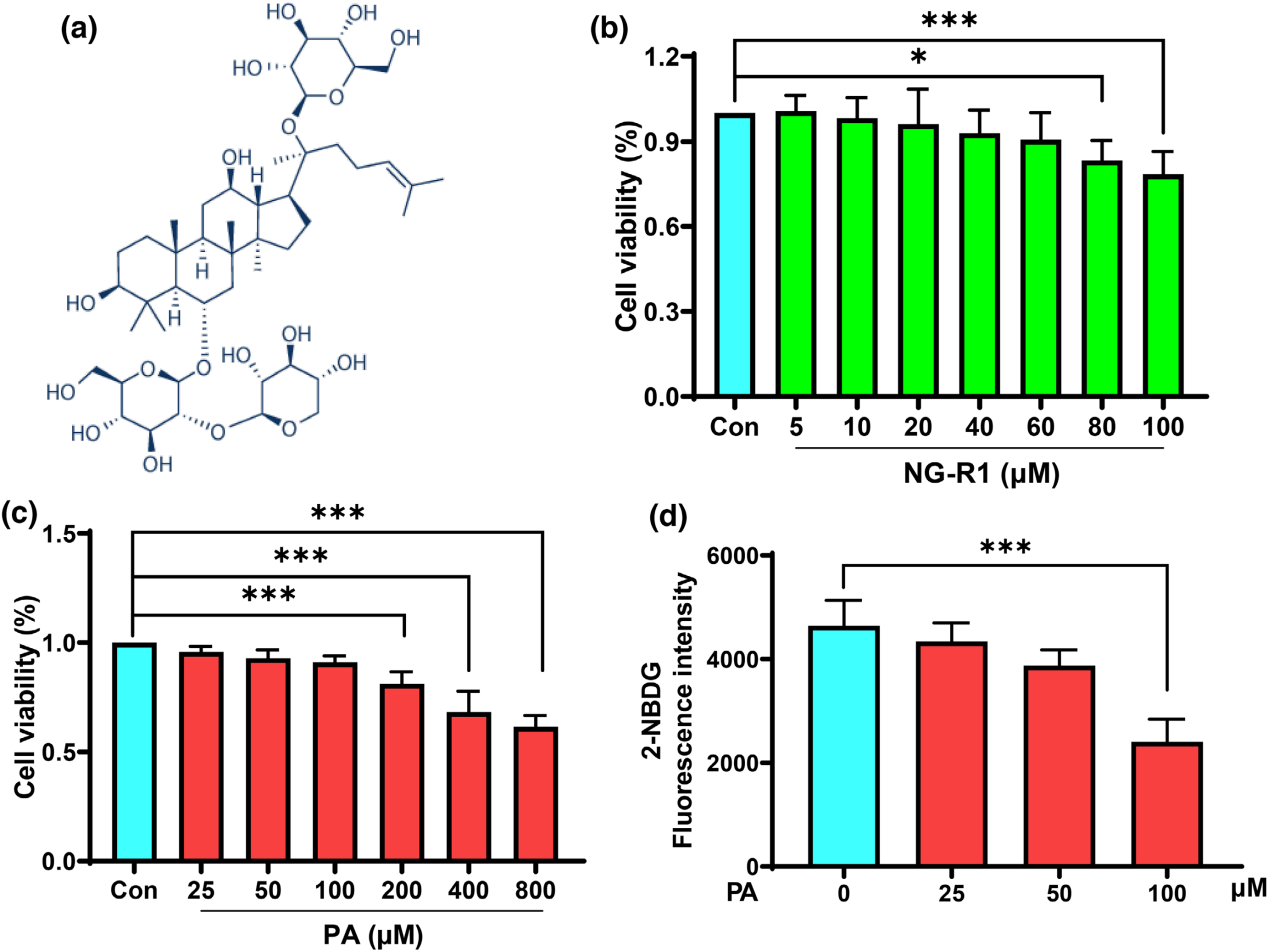
Cytotoxic effect of NG‐R1 or PA on HUVECs and PA‐induced insulin resistance. (a) Chemical structure of NG‐R1. The molecular weight of NG‐R1 is 933 and the molecular formula is C47H80O18. Evaluation of the cytotoxic effects of NG‐R1 (b) or PA (c) on HUVECs were carried out using MTT assay. (d) Optimizing concentration for PA‐induced insulin resistance. HUVECs were exposed PA (25, 50, and 100 μM), then the medium was replaced with 100 nM insulin and 100 μM 2‐NBDG in a serum‐free medium, and incubated for another 30 min. Fluorescence intensity was determined by a fluorescence microplate reader. Data are expressed as mean ± SD. Statistical significance was determined as *p < .05 and ***p < .001, performed using one‐way ANOVA, followed by Tukey's multiple comparisons test.

*Panax notoginseng* (PN) belongs to Araliaceae family (Wang et al., 2016), its roots are often prescribed for maintaining the homeostasis of the microcirculation in the human body and dealing with various disorders in cardiovascular (Liu et al., 2014), neuronal (Xie et al., 2018), and diabetic dysfunctions (Uzayisenga et al., 2014). Notoginsenosides (NGs) are the primary bioactive compounds responsible for the pharmacological effects of *P. notoginseng* (Jin et al., 2019; Xu et al., 2019), with over 20 NGs identified from *P. notoginseng*. Among these NGs, NG‐R1 (Figure 1a) has been demonstrated the most remarkable efficacy, exhibiting diverse biological activities, including antidiabetic and anticancer activity, neuroprotection, and cardiovascular and liver protection (Liu et al., 2020).

Insulin resistance (IR), characterized by reduced responsiveness of peripheral tissues to insulin, represents a pivotal pathophysiological phenotype in metabolic syndrome, such as hypertension, dyslipidemia, nonalcoholic fatty liver disease (NAFLD), and type 2 diabetes (T2D; Beale, 2013). In addition to the liver, adipose tissue, and muscle, accumulated evidence indicates that endothelium plays a crucial role as an insulin target, participating in the development of metabolic insulin resistance provoked by the Western diet (Barrett & Liu, 2013). The vascular endothelium functions as the “first‐responder” to endogenous stimuli, such as nutrients, within the blood, thereby regulating insulin delivery to peripheral tissues and influencing insulin‐mediated glucose disposal in these tissues (Barrett & Liu, 2013). Therefore, insulin resistance in vascular endothelium broadly impacts physiological functions and contributes to metabolic dysregulation. Interestingly, investigations into the time course of insulin resistance development induced by high‐fat feeding have shown that endothelial cell responses precede those observed in other insulin target tissues (Kim et al., 2008). Palmitic acid (PA), a prominent saturated fatty acid and typical ingredient in the Western diet, is known to impair insulin signaling (Fatima et al., 2019). The excessive production of reactive oxygen species (ROS) resulting from lipid metabolism abnormalities further aggravates the burden of the antioxidative system in peripheral metabolic tissues (Onyango, 2018). Therefore, the impairment of the endogenous redox system by high‐fat feeding is critical to the development of insulin resistance.

Several studies demonstrate that naturally derived saponins from *Panax* genus play a role in alleviating insulin resistance (Bai et al., 2018). For example, *P. notoginseng* saponins (PNS) reduced hyperglycemia and insulin resistance of skeletal muscle by upregulating glucose transporter 4 (GLUT4) and activating IRS1‐PI3K/Akt (Guo et al., 2019). Ginsenoside Rg1 (GRg1), a major active saponin in *Panax ginseng*, ameliorates PA‐induced hepatic insulin resistance (Mo et al., 2019). GRg1 reversed PA‐induced reduction of glucose consumption of HepG2 cells by downregulating phosphoenolpyruvate carboxy kinase (PEPCK) and glucose‐6‐phosphatase (G6Pase), and promoted Akt activation but inhibited c‐Jun N‐terminal kinase (JNK) activation. In addition, cellular ROS level elevated in PA‐challenged HepG2 cells was diminished by GRg1 treatment. Additionally, Fan et al. (2019) reported that GRg1 could correct the disorders of glucose and lipid metabolism and improve insulin resistance in rat model induced by high‐fat and high‐sugar diets, possibly due to the suppression of inflammation and glucose output. Another study (Peng et al., 2019) found that rare ginsenosides prevented PA‐induced reduction of pyruvate dehydrogenase (PDH) activity and increased carnitine palmitoyltransferase 1 (CPT1) expression, in addition to ameliorating the uptake and utilization of glucose mediated by insulin in H9c2 cells. This observation indicates that rare ginsenosides exhibit a beneficial effect on myocardial insulin sensitivity under conditions of elevated lipid concentrations. This effect is achieved through the modulation of glucose and fatty acid oxidation pathways, leading to a reduction in mitochondrial oxidative stress.

Nrf2, a member of bZIP transcription factor, plays an essential role in regulating various cytoprotective enzymes, such as NAD(P)H:quinone oxidoreductase 1 (NQO1), heme oxygenase‐1 (HO‐1), superoxide dismutase (SOD), catalase (CAT), and glutathione peroxidase (GPx), in response to oxidative stresses (Bellezza et al., 2018). The activation of Nrf2 can improve insulin sensitivity in the peripheral tissues and prevent the development of insulin resistance (Zhang et al., 2015, 2020). A previous study (Zhai et al., 2018) showed that NG‐R1 administration ameliorated cognitive dysfunctions, insulin resistance, dyslipidemia, inflammation, and oxidative stress by activating Akt/Nrf2/HO‐1 pathway and inhibiting NOD‐like receptor family pyrin domain‐containing 3 (NLRP3) inflammasome in *db/db* mice. In addition, NG‐R1 showed neuroprotection against cerebral ischemia–reperfusion (I/R) injury through estrogen receptor‐dependent activation of Akt/Nrf2/HO‐1 pathway to inhibit NADPH oxidase activity and mitochondrial dysfunction (Fratantonio et al., 2015). These investigations collectively suggest that NG‐R1 functions as a natural agonist for the Nrf2 pathway. Nonetheless, it remains unclear whether NG‐R1 can ameliorate PA‐induced endothelial insulin resistance and exert protective effects on endothelial function through the Nrf2 signaling pathway. Therefore, the objective of this study is to investigate the impact of NG‐R1 on PA‐induced insulin resistance in human umbilical vein endothelial cells (HUVECs) as well as lipid peroxidation and on the activation of the Nrf2 pathway. This study presents new insight into the *P. notoginseng* NG‐R1 prevention of PA‐induced endothelial metabolic disorders and cardiovascular disease (CVD).

## 2 MATERIALS AND METHODS

### 2.1 Materials and chemicals

NG‐R1 (HPLC grade, >97% purity) was supplied from Selleck Chemicals. PA, bovine serum albumin, 3‐(4,5‐dimethylthiazol‐2‐yl)‐2,5‐diphenyltetrazolium bromide (MTT), and insulin were purchased from Sigma‐Aldrich. TRIzol reagent, SybrGreen qPCR master mix, and reverse transcription kit were obtained from Takara Biomedical Technology (Dalian, China). 2‐(*N*‐(7‐Nitrobenz‐2‐oxa‐1,3‐diazol‐4‐yl)amino)‐2‐deoxyglucose (2‐NBDG) was purchased from Thermo Fisher Scientific, Inc. ROS assay kit was purchased from Sigma‐Aldrich. Anti‐KEAP1, anti‐IRS‐1, anti‐phospho‐IRS‐1 (Ser307), anti‐Akt, and anti‐phospho‐Akt (Ser473) antibodies were obtained from Cell Signaling Technology. Anti‐Nrf2 and anti‐phospho‐Nrf2 (Ser40) antibodies were obtained from Abcam. Antibodies against β‐actin and lamin B and horseradish peroxidase (HRP)‐conjugated secondary antibody were purchased from Santa Cruz Biotechnology. All other chemical reagents used were analytical grade.

### 2.2 Cell culture and treatment

This study was approved by the ethics committee of affiliated Jinhua Hospital, Zhejiang University School of Medicine. Human umbilical cords were obtained from full‐term cesarean section surgery in affiliated Jinhua Hospital, Zhejiang University School of Medicine. The patients had been informed a priori and had consented to donate. HUVECs, isolated from freshly human umbilical cords by collagenase digestion of the interior of the umbilical vein, were cultured in gelatin pretreated flasks with medium 199 containing 20% FBS, l‐glutamine (2 mM), HEPES (20 mM), endothelial cell growth supplement (50 μg/mL), penicillin/streptomycin (100 units/mL), and heparin (10 mg/mL). Cells were maintained in a humidified incubator containing 5% CO_2_ at 37°C. The monolayer of HUVECs is characterized by a typical “cobblestone” appearance at confluency under light microscopy. Additionally, CD31 (platelet–endothelial cell adhesion molecule, PECAM) is specifically expressed on endothelial cells as a specific marker for HUVECs. The purity of isolated HUVECs was characterized as 96.7% of CD31 positive cells using flow cytometry. In this study, cells used were from passage 2 to 7.

### 2.3 MTT assay

HUVECs were plated in 96‐well plates coated with 1% gelatin and incubated for 24 h, then treated with NG‐R1 (at 5, 10, 20, 40, 60, 80, and 100 μM) or PA (at 25, 50, 100, 200, 400, and 800 μM) for 48 h. Control cells were treated with PBS only. Following incubation, the medium in each well was replaced with 100 μL fresh medium containing 0.5 mg/mL of MTT and the cells were incubated at 37°C for another 4 h. DMSO (150 μL) was added once the culture medium was removed. Absorbance was recorded at a wavelength of 570 nm by a microplate reader (Infinite F200 Pro; Tecan). Cell viability was expressed as a relative percentage of the nontreated control group.

### 2.4 Glucose uptake assay

HUVECs were plated at 1 × 10^4^/well in 96‐well plates coated with 1% gelatin, and allowed to adhere overnight. Cells were exposed PA (100 μM) for 48 h prepared as described previously [23], then treated with NG‐R1 (10 or 40 μM) for 24 h. Afterward, the medium was removed and replaced with 100 nM insulin and 100 μM 2‐NBDG in a serum‐free fresh medium, and incubated for another 30 min. Subsequently, the cells were washed thrice using PBS and fluorescence intensity was determined at an excitation of 488 nm and an emission of 520 nm using a fluorescence microplate reader (Infinite F200 Pro; Tecan).

### 2.5 Measurement of intracellular ROS level

Intracellular ROS level was determined using ROS assay kit (Sigma‐Aldrich). Briefly, confluent HUVECs were treated with PA (100 μM) in 96‐well plates for 48 h, then treated with NG‐R1 (10 or 40 μM) for 24 h. The HUVECs were incubated with 20 μM 2',7'‐dichlorofluorescin diacetate (DCFDA), a fluorescent probe, at 37°C for 30 min. After three rounds of washing with 1× PBS buffer, fluorescence intensity was determined using a microplate reader (Infinite F200 Pro; Tecan) at an excitation wavelength at 485 nm and an emission wavelength at 535 nm.

### 2.6 Biochemical analysis of lipid peroxidation

To determine the levels of malondialdehyde (MDA) and 4‐hydroxynonenal (4‐HNE), the ratio of reduced glutathione (GSH)/oxidized glutathione (GSSG) and the activity of glutathione peroxidase (GPx), PA‐stimulated HUVECs treated with NG‐R1 were harvested using ice‐cold RIPA containing 1 mM PMSF. The biochemical kits for determining MDA and GSH/GSSG ratio, as well as 4‐HNE ELISA kit were purchased from Sigma‐Aldrich. The analytical kit for GPx activity was purchased from Abcam.

### 2.7 qPCR

Total mRNA was extracted using TRIzol reagent and then cDNA was synthesized by reverse transcription kit (Thermo Fisher Scientific, Inc.). The mRNA levels were quantified using SybrGreen qPCR master mix. The primer pairs used in this study were displayed in Table 1. Amplification and qPCR measurements were performed using the Applied Biosystems 7500 Fast Real‐Time PCR System with the following program: 94°C for 10 min, followed by 40 cycles at 94°C for 10 s, and 60°C for 1 min. The relative expression levels of target genes were calculated using the $2^{-\Delta \Delta C_{t}}$ method.

**TABLE 1:** Designed primer sets for qPCR.

Gene	Primer	5′–3′	Size (bp)
*Ho‐1*	Sense Antisense	CCTTCTTCACCTTCCCCAAC GCCTCTTCTATCACCCTCTG	124
*Nqo1*	Sense Antisense	CAGTTGGGATGGACTTGC CCAGGCAGGATTCTTAATG	101
*Gpx4*	Sense Antisense	GCCTCCCAGTGAGGCAAGAC GGGAAGGCCAGGATCCGCAA	95
*Nrf2*	Sense Antisense	GGTTGGGGTCTTCTGTGG CATTGAGCAAGTTTGGGAG	245
*β‐Actin*	Sense Antisense	CTCTTCCAGCCTTCCTTCCT TCTTCATTGTGCTGGGTGCC	201

### 2.8 Western blotting

To isolate the total cellular protein, cells were homogenized with ice‐cold RIPA lysis buffer and then the lysed samples were centrifuged to remove the insoluble debris. The nuclear and cytoplasmic fractions were isolated using a nuclear and cytoplasmic protein extraction kit (Abcam). The protein concentration in the supernatant was measured using a BCA protein assay kit (Thermo Fisher Scientific, Inc.). Subsequently, 30 μg of protein was separated by sodium dodecyl sulfate–polyacrylamide gel electrophoresis (SDS‐PAGE) and transferred to PVDF membranes (Millipore). The membranes were blocked with 5% nonfat dry milk (dissolved in TBST) for 1 h followed by overnight incubation at 4°C with specific primary antibodies, including KEAP1 (Cell Signaling Technology, 4678 1:1000 dilution), IRS‐1 (Cell Signaling Technology, 3194, 1:1000 dilution), phospho‐IRS‐1 Ser307 (Cell Signaling Technology, 2381, 1:1000 dilution), Akt (Cell Signaling Technology, 9272, 1:1000 dilution) and phospho‐Akt Ser473 (Cell Signaling Technology, 4060, 1:2000 dilution), Nrf2 (Abcam, ab62352, 1:1000 dilution), anti‐phospho‐Nrf2 Ser40 (Abcam, ab76026, 1:2000 dilution), β‐actin (Santa Cruz, sc‐47778, 1:1000 dilution), and lamin B (Santa Cruz, sc‐374015, 1:1000 dilution). Then, the appropriate HRP‐conjugated secondary antibodies were applied for 1 h at room temperature. Ultimately, the signals were visualized with a chemiluminescence system (Bio‐Rad). Quantitative analysis was performed by ImageJ.

### 2.9 Co‐immunoprecipitation

Cell lysates from HUVECs were incubated with antibodies against Nrf2 or KEAP1 for 1 h, followed by incubating with protein A/G beads (Sigma‐Aldrich) for 1 h to precipitate the immunocomplex. Then, the immunocomplex was stripped by boiling with 5× loading buffer for immunoblotting incubated with indicated antibodies.

### 2.10 Molecular docking

Molecular docking was performed using AutoDock vina 1.1.2 package to investigate the potential binding of NG‐R1 to KEAP1. The crystal structure of KEAP1 (6LRZ) was obtained from RCSB Protein Data Bank, while the structure of NG‐R1 was obtained from PubChem. The simulation protein of KEAP1 for molecular docking was prepared by removing water molecules and ligands. The most stable docking model was selected based on conformation of best score predicted by the AutoDock due to the lowest docking energy, and further analyzed using PyMoL 2.4.0 software.

### 2.11 Nrf2 gene silencing

HUVECs were cultured in six‐well plates to reach 70% confluence, then the medium was replaced with OPTI‐MEM (Gibco). Cells were transfected with 10 nM siRNA against Nrf2 (Nrf2 Human siRNA Oligo Duplex; Origene) or control siRNA in OPTI‐MEM containing 10% FBS without antibiotics using lipofectamine RNAiMAX Transfection Reagent (Invitrogen). After incubation for 48 h, the expression level of Nrf2 was determined by western blotting.

### 2.12 Statistical analysis

Statistical differences were analyzed with one‐way ANOVA followed by a post hoc Tukey's test among groups using SPSS software (v21.0). The *p* values < .05 were considered statistically significant.

## 3 RESULTS

### 3.1 NG‐R1 ameliorated PA‐induced insulin resistance

**FIGURE 2:**
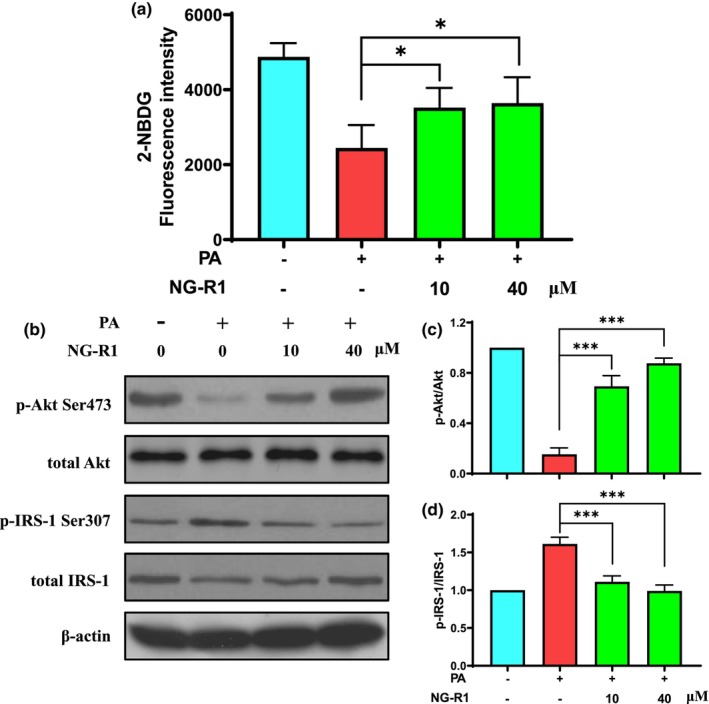
NG‐R1 ameliorates glucose uptake and insulin resistance in PA‐treated HUVECs. (a) HUVECs were exposed to 100 μM PA for 48 h and then treated with NG‐R1 for 24 h. Culture medium was replaced with 100 nM insulin and 100 μM 2‐NBDG in a serum‐free fresh medium, and then incubated for 30 min. Fluorescence intensity was measured using a fluorescence microplate reader. (b) Protein levels of p‐Akt Ser473, total Akt, p‐IRS‐1 Ser307, and total IRS‐1 were determined by western blotting. (c) Ratio of p‐Akt Ser473 to total Akt. (d) Ratio of p‐IRS‐1 Ser307 to total IRS‐1. Data are expressed as mean ± SD. Statistical significance was determined as *p < .05 and ***p < .001, performed using one‐way ANOVA, followed by Tukey's multiple comparisons test.

NG‐R1 revealed no toxic effect on the viability of HUVECs for 48 h at different concentrations up to 60 μM (Figure 1b). When the concentration of NG‐R1 increased to ≥80 μM, cell viability of HUVECs was significantly decreased compared to the control group. Therefore, 10 and 40 μM of NG‐R1 concentrations were chosen for this study, considering the concentrations achievable in plasma under normal circumstance. Moreover, cell viability was significantly (*p* < .05) inhibited by the treatment with a high dose (≥200 μM) of PA for 48 h (Figure 1c). For cellular model of PA‐induced insulin resistance, we tried three PA concentrations (25, 50, and 100 μM) and found that the glucose uptake of HUVECs significantly (*p* < .05) decreased after 100 μM of PA treatment as demonstrated by the decrease in fluorescence density in HUVECs (Figure 1d). Impaired glucose uptake triggered by PA treatment was significantly (*p* < .05) improved by NG‐R1 treatment at both 10 and 40 μM concentrations as shown in Figure 2a. In addition, PA induced a decrease in the level of phosphorylated Akt at Ser473 (Figure 2b,c) and an increase in the level of phosphorylated IRS‐1 at Ser307 (Figure 2b,d), while NG‐R1 (10 and 40 μM) significantly (*p* < .05) increased Akt phosphorylation as well as significantly (*p* < .05) decreased the phosphorylated IRS‐1. These findings confirm that NG‐R1 enhanced the insulin sensitivity of PA‐treated HUVECs.

### 3.2 NG‐R1 attenuates PA‐induced ROS production and lipid peroxidation

**FIGURE 3:**
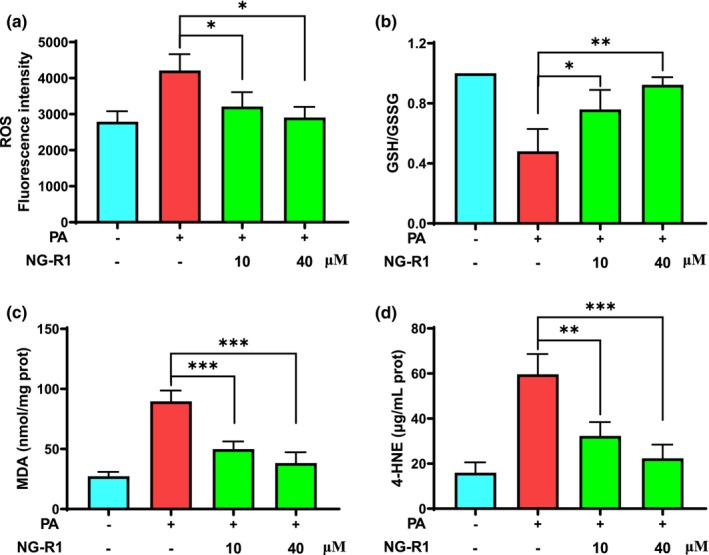
NG‐R1 alleviates PA‐induced redox imbalance. (a) Intracellular ROS level was evaluated using ROS assay kit. HUVECs were treated with PA for 48 h and then with NG‐R1. The HUVECs were incubated with a DCFDA fluorescent probe and fluorescence intensity measured using a microplate reader. (b) GSH/GSSG ratio, (c) MDA, and (d) 4‐HNE were determined using their respective detection kits according to the manufacturer's instructions. Data are expressed as mean ± SD. Statistical significance was determined as *p < .05, **p < .01, and ***p < .001, performed using one‐way ANOVA, followed by Tukey's multiple comparisons test.

Oxidative stress injury in HUVECs is the main cause of insulin resistance induced by high‐fat diets or obesity, and therefore, the ROS level and GSH/GSSG ratio were determined to investigate whether those pathological process could be alleviated by NG‐R1. PA increased ROS level in HUVECs, whereas NG‐R1 at both 10 and 40 μM significantly (*p* < .05) reduced ROS production (Figure 3a). Additionally, the GSH/GSSG ratio, also an essential index of redox status, was remarkably reduced by approximately 52% in PA‐treated HUVECs, while both concentrations of NG‐R1 strongly inhibited this reduction (Figure 3b). To investigate the effect of oxidative stress on lipid peroxidation, the levels of MDA and 4‐HNE in PA‐treated HUVECs were measured. MDA level was significantly higher in PA‐treated HUVECs (89.6 nmol/mg protein) than in nontreated HUVECs (27.3 nmol/mg protein). In contrast, NG‐R1 significantly (*p* < .05) inhibited the increase of MDA level triggered by PA treatment (Figure 3c). 4‐HNE is one of the most cytotoxic products of lipid peroxidation. PA treatment significantly increased the level of 4‐HNE in HUVEC, whereas NG‐R1 significantly reduced the level of 4‐HNE (Figure 3d). In summary, these results suggest that NG‐R1 protects HUVECs against PA‐induced lipid peroxidation.

### 3.3 NG‐R1 increases antioxidant enzymes in HUVECs and activates Nrf2 signaling

**FIGURE 4:**
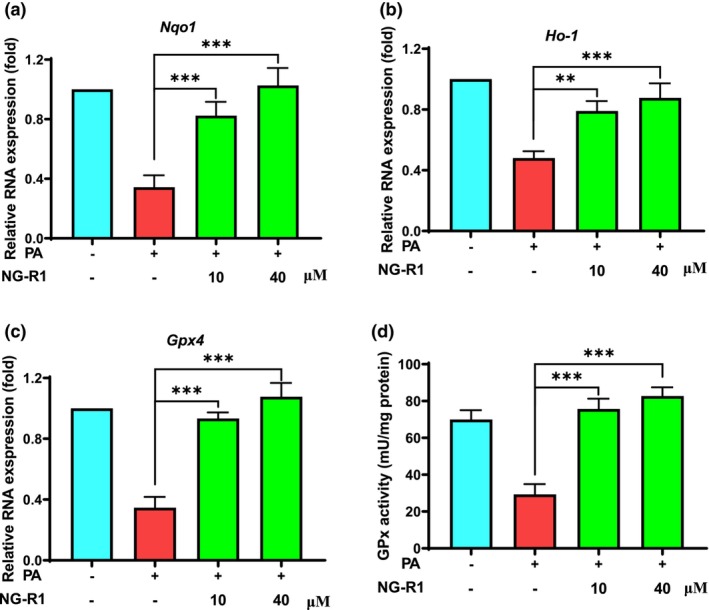
NG‐R1 upregulates PA‐induced reduction of antioxidant enzymes. qPCR analysis of representative genes encoding antioxidant enzymes (a) *Nqo1*, (b) *Ho‐1*, and (c) *Gpx4*. (d) GPx activity was determined by commercial analytical kit. Data are expressed as mean ± SD. Statistical significance was determined as **p < .01 and ***p < .001, performed using one‐way ANOVA, followed by Tukey's multiple comparisons test.

**FIGURE 5:**
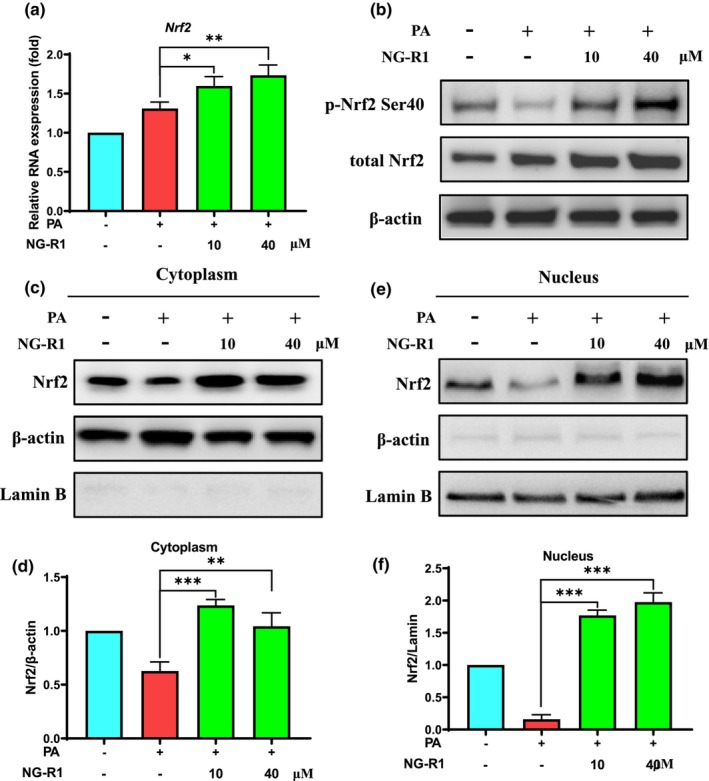
NG‐R1 activates Nrf2 signaling by promoting nuclear entry of Nrf2. (a) qPCR analysis of Nrf2 expression. (b) The protein levels of total Nrf2 and phosphorylated Nrf2. (c) Protein level of Nrf2 in the cytoplasm of HUVECs was determined by western blotting and (d) quantitative analysis of the densitometry of each band was performed using ImageJ software. (e) Protein level of Nrf2 in the nucleus and (f) quantitative analysis. Data are expressed as mean ± SD. Statistical significance was determined as *p < .05, **p < .01, and ***p < .001, performed using one‐way ANOVA, followed by Tukey's multiple comparisons test.

Multiple antioxidant enzymes, such as NAD(P)H:quinone oxidoreductase 1 (NQO1), heme oxygenase‐1 (HO‐1), and glutathione peroxidase (GPx), are essential for the redox balance in HUVECs. PA treatment significantly reduced the mRNA levels of *Nqo1* (Figure 4a), *Ho‐1* (Figure 4b), and *Gpx4* (Figure 4c) in HUVECs, while NG‐R1 treatment at both 10 and 40 μM significantly (*p* < .05) upregulated the expression of those genes, as well as enhanced GPx activity (Figure 4d). Nrf2 is an important transcription factor responsible for antioxidant enzymes. Interestingly, PA treatment increased the mRNA (Figure 5a) and total protein (Figure 5b) level of Nrf2, but did not increase its phosphorylation levels (Figure 5b), suggesting that PA probably blocked Nrf2 phosphorylation and subsequent signaling transduction by inhibiting kinase upstream of Nrf2. While, NG‐R1 (10 and 40 μM) not only upregulated the expression both at transcript (Figure 5a) and total protein (Figure 5b) levels, but also increased levels of Nrf2 phosphorylation (Figure 5b). Then, the expression levels of Nrf2 both in cytoplasmic and nuclear fractions were analyzed. NG‐R1 increased Nrf2 in the cytoplasm of HUVECs (Figures 5c,d), it also promoted Nrf2 to enter or remain in the nucleus (Figures 5e,f). This suggests that NG‐R1 stimulated the activation of Nrf2 signaling and then initiated the expression of antioxidant enzymes in PA‐treated HUVECs. Overall, NG‐R1 reversed the endothelial oxidative stress triggered by PA.

**FIGURE 6:**
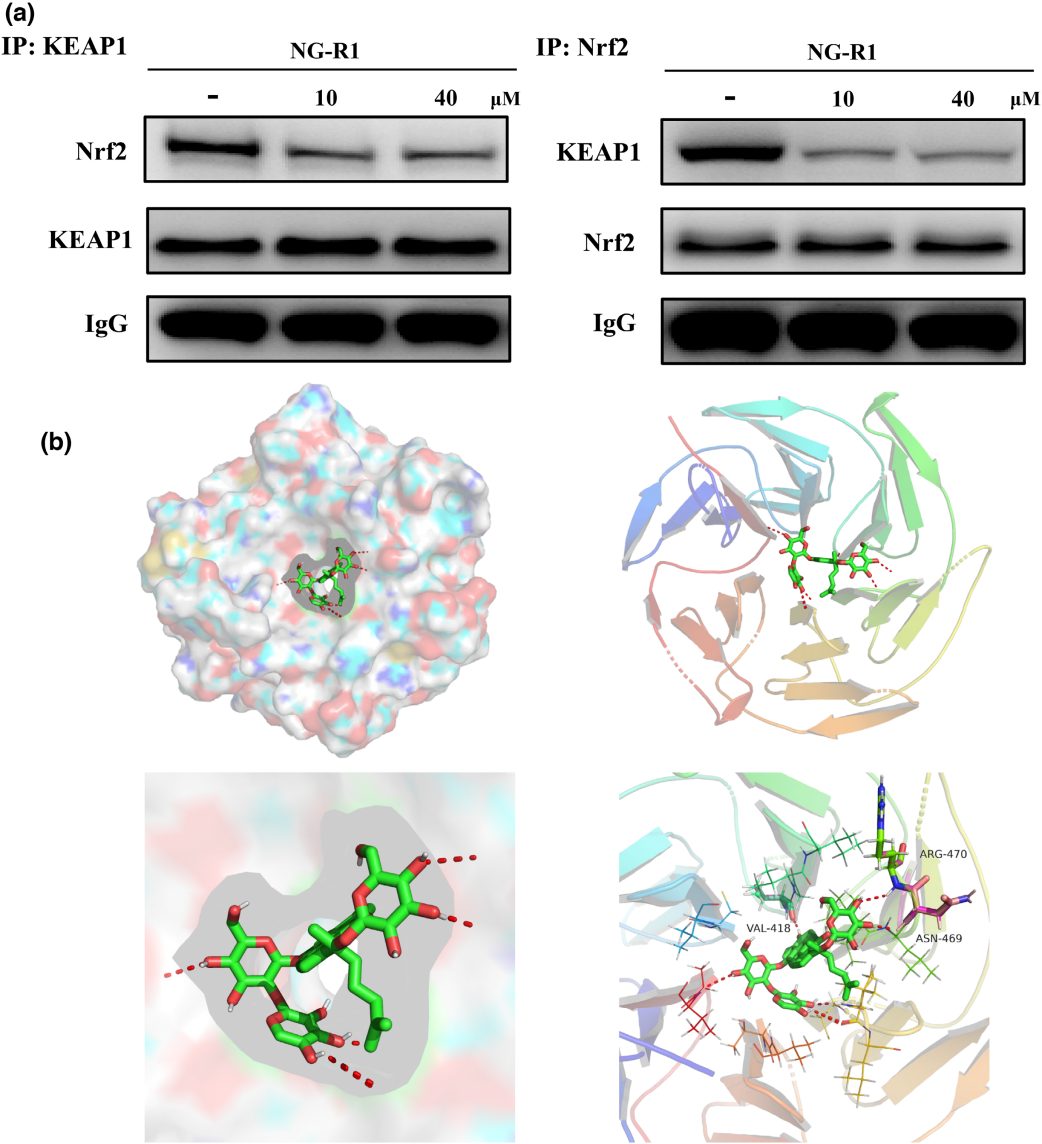
NG‐R1 diminishes the interaction between Nrf2 and KEAP1. (a) Co‐immunoprecipitation. HUVECs were treated with or without NG‐R1, and then lysates were immunoprecipitated with the anti‐Nrf2 or anti‐KEAP1 antibodies, followed by western blotting. (b) Molecular docking of NG‐R1 with Kelch domain of KEAP1.

KEAP1 tightly interacts with Nrf2, which will mediate the degradation of Nrf2 and block its entry into the nucleus to initiate the expression of antioxidant gene. Our present data from co‐immunoprecipitation (Figure 6a) showed that NG‐R1 inhibited the binding of Nrf2 and KEAP1. Furthermore, the molecular docking demonstrated the theoretical three‐dimensional binding mode of NG‐R1 and the Kelch domain of KEAP1 with the lowest docking energy (Figure 6b). NG‐R1 was positioned at the hydrophobic pocket of KEAP1, surrounded by the residues Val‐418, Asn‐469, and Agr‐470, forming a stable hydrophobic binding that enabled NG‐R1 anchor to KEAP1. Additionally, the estimated binding energy of NG‐R1 and KEAP1 complex was −7.75 kcal/mol. Thus, NG‐R1 activates the Nrf2/ARE signaling potentially through antagonizing with KEAP1, thereby enhancing antioxidant capacity in PA‐treated HUVECs.

### 3.4 Nrf2 silencing diminishes NG‐R1‐induced glucose uptake and insulin sensitivity

**FIGURE 7:**
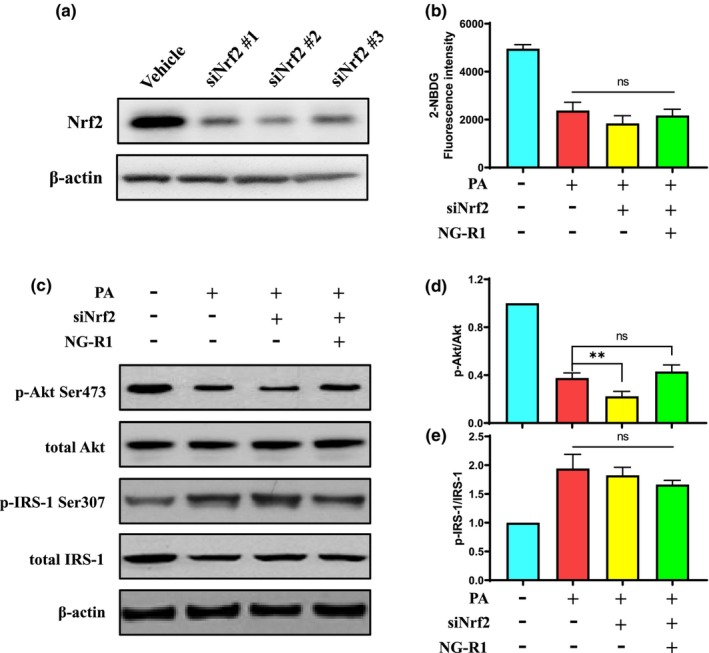
NG‐R1 alleviates PA‐induced insulin resistance depending on Nrf2 signaling. (a) siRNA knockdown of Nrf2 expression in HUVECs. Nrf2 protein expression was determined by western blotting after transfection with siRNA. (b) Glucose uptake, indicated by fluorescence intensity, was measured using a fluorescence microplate reader. (c) Protein levels of p‐Akt Ser473, total Akt, p‐IRS‐1 Ser307, and total IRS‐1 were determined by western blotting. (d) Ratio of p‐Akt Ser473 to total Akt. (e) Ratio of p‐IRS‐1 Ser307 to total IRS‐1. Data are expressed as mean ± SD. Statistical significance was determined as **p < .01 and ns, no significance, performed using one‐way ANOVA, followed by Tukey's multiple comparisons test.

To determine if NG‐R1 alleviated PA‐induced insulin resistance through Nrf2 signaling, Nrf2 was silenced using siRNA before NG‐R1 treatment (Figure 7a). PA‐impaired glucose uptake of HUVECs could not be alleviated by NG‐R1 without the Nrf2 expression (Figure 7b). We also found that NG‐R1 could not alleviate PA‐impaired insulin signaling in HUVECs with altered Nrf2 expression. This was proved by the suppressed Akt phosphorylation level (Figure 7c,d) and enhanced IRS‐1 phosphorylation level (Figure 7c,e), which was not affected by NG‐R1 treatment. In summary, these results suggest that the protective effect of NG‐R1 against PA‐induced insulin resistance in HUVECs is directly dependent on the Nrf2/ARE pathway.

## 4 DISCUSSION

As the global increase of obesity rate, there is an acceleration of the incidence and prevalence of insulin resistance along with associated CVD (Hill et al., 2021). Chronic rise in plasma free fat acids (FFAs) has been reported to evoke the development of insulin resistance and endothelial dysfunction especially in diabetic or obese individuals, thereby contributing to the cardiovascular risk (Hsueh & Law, 2003). Mounting evidence demonstrate that treatment of endothelial cells with FFAs impairs insulin signaling due to the production of ROS, which subsequently triggers the activation of JNK or inhibition of IκB kinases (IKK), ultimately leading to the vascular endothelial dysfunction (Batumalaie et al., 2016; Inoguchi et al., 2000). In addition, accumulated evidence indicate the pivotal role of insulin resistance in the pathogenesis of CVD, such as atherosclerosis (Hill et al., 2021). Specifically, insulin‐mediated production of nitric oxide (NO) from the vascular endothelium is known to enhance blood flow and promote glucose disposal (Ormazabal et al., 2018). Conversely, diminished sensitivity of vascular endothelium to insulin, particularly reduced NO production, contributes to the development of CVD in the context of insulin resistance. Consequently, interventions aimed at reversing the insulin resistance status of endothelial cells present promising avenues for addressing metabolic syndrome and CVD therapeutically, offering novel insights into potential treatment strategies.

Numerous studies have consistently demonstrated that the activation of redox‐sensitive genes downstream of the Nrf2/ARE pathway is highly associated with response to redox imbalance and xenobiotics (Bellezza et al., 2018). Under normal conditions, Nrf2 is sequestered in the cytoplasm through its binding to the repressor protein KEAP1. However, in the presence of chemical or oxidative stress, Nrf2 can evade KEAP1 and translocate into the nucleus, where it initiates the expression of antioxidant genes. The accumulation of evidence strongly supports the hypothesis that pharmacologically induced expression of cytoprotective proteins regulated by Nrf2/ARE pathway may contribute to an atheroprotective and anti‐inflammatory phenotype in vascular endothelium (Chen et al., 2015; Mimura & Itoh, 2015). NG‐R1, a saponin isolated from *P. notoginseng*, exhibits a variety of bioactivities, including antioxidative, anti‐inflammatory, and antiapoptotic (Liu et al., 2014). Additionally, NG‐R1 could ameliorate insulin resistance, hyperinsulinemia, dyslipidemia, and inflammation in *db/db* mice and markedly decreased hyperglycemia‐induced oxidative stress in hippocampal neurons by activating the Akt/Nrf2 pathway and inhibiting NLRP3 inflammasome, demonstrating an excellent neuroprotective effect (Zhai et al., 2018). In the insulin signaling pathway, the activated Akt (phosphorylated at Ser473) is able to catalyze the phosphorylation of AS160 substrate protein, leading to the translocation of GLUT from the cytoplasmic vesicles onto the cell membrane, thereby enhancing the insulin‐dependent uptake of glucose (Beg et al., 2017). Furthermore, the phosphorylation of IRS‐1 at Ser307 attenuates insulin signaling and contributes to insulin resistance (Rui et al., 2001). In this study, NG‐R1 effectively alleviates PA‐impaired glucose uptake in HUVECs, increases the Akt phosphorylation at Ser473, and reduces the IRS‐1 phosphorylation at Ser307, suggesting that NG‐R1 enhances the insulin sensitivity of PA‐treated HUVECs.

In this study, we observed an elevation in oxidative stress in PA‐treated HUVECs, as indicated by a decrease in glutathione reduced/oxidized ratio (GSH/GSSG), an increase in MDA level, and enhanced protein modification by 4‐HNE. GSH and GSSG serve as the principal redox buffers of HUVECs, with GSH acting as a vital protective antioxidants and ROS scavengers. The ratio of total GSH to GSSG is a crucial biological index for assessing oxidative damage (Leskova et al., 2017). MDA, a toxic metabolite of lipid peroxidation, is considered as a biomarker for the onset of metabolic diseases (Piconi et al., 2003). Additionally, 4‐HNE, a highly reactive end product of lipid peroxidation, can react with various macromolecular substances, particularly proteins containing cysteine, lysine, and histidine residues (Chapple et al., 2013). This protein modification, referred to as HNE–protein adduction, contributes to protein cross‐linking and impairs their activities. The inadequate capacity for scavenging free radicals is a significant factor contributing to insulin resistance. Notably, recent reports have demonstrated that NG‐R1 regulates the cellular redox state and protects capillary endothelial cells against high glucose‐induced oxidative damage (Fan et al., 2017). This protection is evidenced by increased concentrations of cellular NAD^+^, NADPH, and GSH, increased ratios of NAD^+^/NADH, NADPH/NADP^+^, and GSH/GSSG, increased activity of CAT, reduced production of ROS and nitrotyrosine, and decreased activities of NADPH oxidase and poly ADP‐ribose polymerase (PARP).

**FIGURE 8:**
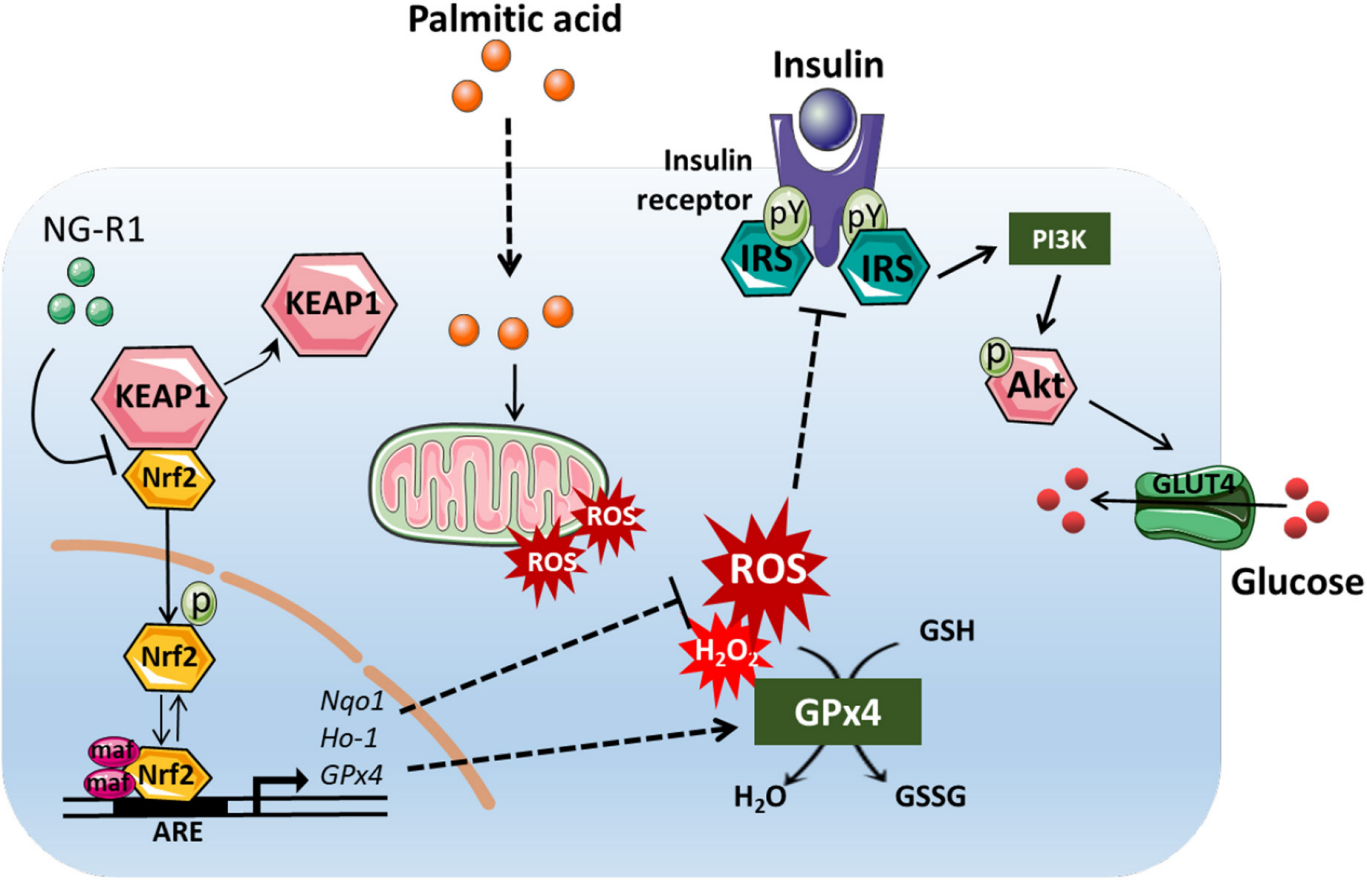
Proposed model for the potential mechanism of ameliorating PA‐induced insulin resistance in HUVECs by NG‐R1.

Nrf2 serves as a central regulator of antioxidant and detoxification enzymes (Feng et al., 2020). Dysfunction of Nrf2/ARE signaling pathway is closely associated with the development of insulin resistance and metabolic disorders, as there exists a strong link between redox imbalance and insulin resistance (Zhang et al., 2020). Activation of Nrf2 represents a potential therapeutic intervention against oxidative stress in the endothelial cells and vascular diseases. NG‐R1 has been shown to be an agonist of Nrf2 signaling pathway in previous studies. For example, NG‐R1 protects HK‐2 cells from AGEs‐induced mitochondrial damage and ROS production via upregulation of the Nrf2/HO‐1 pathway (Zhang et al., 2019). Similarly, our current findings indicate that NG‐R1 activates Nrf2 signaling pathway, enhancing the expression of antioxidant enzymes (HO‐1, NQO1, and GPX4) to scavenge ROS, and increasing reduced glutathione levels to eliminate the lipid peroxidation products. Silencing *Nrf2* reversed the protective effect of NG‐R1 on PA‐treated HUVECs, as evidenced by the impaired glucose uptake and phosphorylation of Akt and IRS‐1. Overall, NG‐R1 boosts the expression of antioxidant genes to facilitate free radical scavenging and ameliorates PA‐induced insulin resistance in HUVECs, potentially through Nrf2/ARE signaling pathway (as illustrated in Figure 8). This study provides novel insights into potential treatments for metabolic diseases and CVD.

Initial research attempts to treat metabolic diseases was through exogenous antioxidant supplements, such as vitamin E, to lessen oxidative stress (Winklhofer‐Roob et al., 2003), however, the protective effect of exogenous antioxidants is unsatisfactory. A promising approach to reducing oxidative stress involves enhancing endogenous antioxidant defense systems instead of depending on exogenous antioxidant supplements. NG‐R1 supplementation in human subjects has been deemed safe, as no adverse side effects have been reported to date. Additionally, there are existing studies that have investigated the pharmacokinetic properties of ginsenosides in humans (Duan et al., 2018; Tawab et al., 2003; Wang et al., 2011). For example, a study (ChiCTR‐ONC‐09000603; www.chictr.org) characterized pharmacokinetics and metabolism of ginsenosides from an orally ingested extract of *P. notoginseng* roots (1:10, water:extract) was carried out and analyzed using liquid chromatography–mass spectrometry (Hu et al., 2013). In vivo studies and clinical trials are required to determine if these results are translatable to vascular endothelium (Shao et al., 2020).

## 5 CONCLUSION

NG‐R1 is a bioactive compound derived from *P. notoginseng*, known for its remarkable cardiovascular health‐promoting properties. In this study, NG‐R1 effectively alleviated impaired glucose uptake, restored Akt phosphorylation, and significantly reduced IRS‐1 phosphorylation at Ser307, indicating an enhancement in insulin sensitivity of PA‐treated HUVECs. Furthermore, NG‐R1 alleviated PA‐induced lipid peroxidation and enhanced the activation of Nrf2 signaling. Thus, the findings of this study demonstrate that NG‐R1 effectively mitigates insulin resistance induced by PA by reducing oxidative stress through the activation of the Nrf2/ARE pathway. These results suggest that NG‐R1 holds promise as a potential functional food additive for addressing metabolic disorders and CVD.

## AUTHOR CONTRIBUTIONS

**Jingjing Wang:** Data curation (lead); formal analysis (lead); investigation (lead); methodology (lead); software (lead); writing – original draft (lead). **Xun He:** Data curation (equal); formal analysis (equal); investigation (equal); methodology (equal); software (lead); writing – original draft (equal). **Shiwen Lv:** Conceptualization (lead); funding acquisition (lead); project administration (lead); resources (lead); supervision (lead); validation (equal); visualization (lead); writing – review and editing (lead).

## CONFLICT OF INTEREST STATEMENT

The authors declare that the research was conducted in the absence of any commercial or financial relationships that could be construed as a potential conflict of interest.

## Data Availability

The data that support the findings of this study are available from the corresponding author on request.
